# IKBKE modulates autophagy and progestin resistance in endometrial cancer

**DOI:** 10.3389/fonc.2025.1590966

**Published:** 2025-11-25

**Authors:** Jiahui Wang, Xianchao Kong

**Affiliations:** Department of Gynaecology, The Second Affiliated Hospital of Harbin Medical University, Harbin, China

**Keywords:** IKBKE, endometrial cancer, progestin-resistance, autophagy, medroxyprogesterone acetate (MPA)

## Abstract

**Background/objectives:**

Endometrial cancer (EC) is a prevalent malignancy in women, with up to 16% of cases diagnosed in individuals under 40 years old. Progestin-based therapies are essential for fertility preservation in EC patients, but resistance to these treatments remains a major challenge. IKBKE, an oncogenic kinase implicated in various cancers, including breast, ovarian, and prostate, has an unclear role in autophagy regulation and progestin resistance in EC. This study aims to investigate the involvement of IKBKE in these processes.

**Methods:**

A progestin-resistant EC cell line was established to assess the effects of IKBKE knockdown and treatment with CYT387, a selective IKBKE inhibitor. *In vitro* assays, including MTT viability, wound healing, colony formation, and Transwell invasion assays, were performed to evaluate cell proliferation, migration, and invasion. Autophagic activity was analyzed following CYT387 treatment.

**Results:**

IKBKE knockdown significantly reduced cell proliferation, migration, in progestin-resistant EC cells. CYT387 treatment inhibited autophagic activity and decreased cell viability in these cells.

**Conclusions:**

These findings highlight the crucial role of IKBKE in regulating autophagy and mediating progestin resistance in EC. This study provides new insights into IKBKE as a potential molecular target that contributes to understanding the mechanisms underlying progestin resistance in endometrial cancer.

## 1 Introduction

Endometrial cancer (EC) is a common malignancy among females, with its prevalence rising among premenopausal women. One study reported that 44.8% of diagnosed cases were in premenopausal women, and up to 16% of cases were diagnosed in women under 40 (1). Hormonal therapy, particularly progestin treatment, is a key therapeutic strategy for patients with advanced or recurrent EC, as well as for young patients who wish to preserve fertility (2–4). However, progestin resistance affects approximately 30% of younger patients with EC, regardless of medication or treatment protocol, limiting treatment efficacy and worsening survival outcomes (5, 6). Therefore, understanding the molecular mechanisms underlying progestin resistance in EC is critical for identifying new therapeutic targets to enhance treatment efficacy.

IKBKE (inhibitor of nuclear factor kappa-B kinase subunit epsilon) is an oncogenic kinase implicated in multiple cancers, including breast, ovarian, lung, and prostate cancer (7–12). It promotes tumor cell proliferation, survival, and immune evasion through key signaling pathways such as NF-κB, AKT, and STAT3 (13–17). IKBKE overexpression is linked to chemotherapy resistance, particularly to cisplatin and paclitaxel in breast and ovarian cancers, as well as resistance to EGFR tyrosine kinase inhibitors in non-small cell lung cancer (18–21). Given its role in therapeutic resistance, IKBKE represents a potential target for overcoming drug resistance. However, its involvement in progestin-resistant EC remains unclear.

Autophagy is a conserved process that maintains cellular homeostasis by degrading and recycling dysfunctional organelles and proteins (21). Dysregulated autophagy under pathological conditions can contribute to disease progression (22–24). IKBKE has been identified as a key regulator of autophagy in various cancer models, modulating vesicle formation and autophagy-related proteins such as Beclin-1 and LC3 through its aberrant expression (17, 25, 26). IKBKE inhibition disrupts autophagy, leading to reduced cancer cell proliferation and increased apoptosis (19, 20). In low-grade glioma, IKBKE regulates autophagy and contributes to disease progression through pathways such as PI3K/AKT/mTOR. It also serves as a prognostic marker, with its expression effectively predicting survival differences ranging from 2 to 10 years (27). However, the relationship between IKBKE and autophagy in EC remains to be explored.

CYT387, also known as momelotinib, is a multi-target JAK/STAT inhibitor (28). In addition to its role in inhibiting JAK/STAT signaling, preclinical studies demonstrated the efficacy of CYT387 in triple-negative breast cancer, where it suppressed tumor growth by inhibiting IKBKE-induced NF-κB and STAT activation (29). Therefore, CYT387 has also been identified as a potent IKBKE inhibitor that effectively blocks NF-κB-driven tumor proliferation, survival, and immunomodulation. Given the role of IKBKE in autophagy and drug resistance, CYT387 serves as a useful tool to investigate the functional impact of IKBKE inhibition in progestin-resistant EC.

In this study, we demonstrate that IKBKE is upregulated in progestin-resistant EC cells and promotes their proliferation and migration. IKBKE knockdown suppresses tumor growth and reduces autophagy, highlighting its role in autophagy-mediated progestin resistance. Furthermore, IKBKE inhibition with CYT387 overcomes progestin resistance. Collectively, these findings provide new insights into the therapeutic potential of targeting IKBKE-mediated autophagy in EC.

## 2 Materials and Methods

### 2.1 Cell culture

Human EC Ishikawa cells were obtained from Procell Life Science & Technology Co., Ltd. (Wuhan, China). Cells were cultured in Dulbecco’s Modified Eagle Medium (Thermo Fisher Scientific) supplemented with 10% fetal bovine serum and incubated in a 5% CO_2_ environment at 37 °C.

### 2.2 Establishment of progestin-resistant EC cell lines

To induce progestin resistance, progestin-sensitive EC cells were exposed to medroxyprogesterone acetate (MPA, CDAA-S-590045D3, Sigma-Aldrich Co., St. Louis, MO, USA) for 6 months. MPA concentration was doubled monthly until reaching 10 μM (30), and the final concentration of DMSO in all drug treatment groups during the experiment was standardized to 0.1%. The Ishikawa cell-specific culture medium containing MPA was replaced every three days. When cells reached 90% confluence, viable cells were passaged and subsequently cultured in 10 μM MPA. Cell proliferation and relevant signaling pathways were assessed following drug selection.

### 2.3 RT-qPCR

Total RNA was isolated from samples and quantified to ensure equal input (1 µg per reaction). Genomic DNA was removed using 5×gDNA Digester Mix (Yeasen, China), and reverse transcription was carried out with the Hifair^®^ III SuperMix Plus kit (Yeasen, China) according to the manufacturer’s protocol. Gene-specific primers were dissolved and diluted to working concentrations using nuclease-free water and stored appropriately. Quantitative PCR was performed using the Hieff^®^ qPCR SYBR Green Master Mix (No Rox, Yeasen, China) in a 20 µl reaction volume under two-step cycling conditions. Relative gene expression was analyzed using the 2^–ΔΔCt method, with normalization to internal control gene GAPDH. The primer sequences used were: IKBKE forward 5’-GAGAAGTTCGTCTCGGTCTATGG-3’ and reverse 5’-TGCATGGTACAAGGTCACTCC-3’; GAPDH forward 5’-GGAGCGAGATCCCTCCAAAAT-3’ and reverse 5’-GGCTGTTGTCATACTTCTCATGG-3’; MDR1 forward 5’-GGAGCCTACTTGGTGGCACATAA-3’ and reverse 5’-TGGCATAGTCAGGAGCAAATGAAC-3’; PR forward 5’-CCACCATCCACTACAACTACAT-3’ and reverse 5’-AAACACGCACCTCAAAGC-3’.

### 2.4 Western blot

Cells were lysed on ice for 30 minutes using lysis buffer with phenylmethylsulfonyl fluoride and centrifuged to collect protein extracts. A total of 30 μg protein was separated by 15% SDS-PAGE and transferred onto polyvinylidene difluoride membranes. The membranes were blocked at room temperature for 15 minutes using a rapid blocking solution (Wuhan Sevier Biotechnology Co., Ltd., Wuhan, China). Subsequently, membranes were incubated for 16 hours at 4 °C with primary antibodies against progesterone receptor (PR, 5264; Zen Bio, Chengdu, China), IKBKE (3416; Cell Signaling Technology), NF-κB p65 (8242; Cell Signaling Technology), phosphorylated NF-κB p65 (Ser536; 3033; Cell Signaling Technology), LC3B (3868; Cell Signaling Technology), ATG5 (2630; Cell Signaling Technology), Beclin-1 (3495; Cell Signaling Technology), P62 (5114; Cell Signaling Technology), MDR 1 (13342; Cell Signaling Technology), and β-actin (ac-026; ABclonal, China). Membranes were then incubated with rabbit anti-mouse IgG secondary antibodies conjugated with specific horseradish peroxidase for 1 hour at room temperature. Protein bands were visualized using an enhanced chemiluminescence kit (Pierce, Rockford, IL, USA).

### 2.5 Transfection

Adenovirus carrying IKBKE shRNA (HYKY-230307016-DLV, OBiO Technology, Shanghai), viral titer 3×10^8^ PFU/mL. Cells were seeded into 24-well culture plates and trypsinised for quantification. The cell suspension density was standardized to 5×10^4^ cells/well to achieve 30–40% confluence at viral transduction. Lentiviral particles were added after replacing the medium with fresh culture solution when target confluence was attained. Twelve hours post-transduction, the medium was completely replaced to maintain cell viability. After 72 hours of incubation, puromycin selection was applied to isolate stable clones. Viral expression efficiency was assessed over time using qPCR, western blotting, or fluorescence-activated cell sorting with target-specific antibodies.

### 2.6 Cell proliferation assay

The MTT assay was performed to evaluate cell viability and proliferation. Briefly, the selected cell lines were cultured in complete medium containing an appropriate concentration of serum. Cells were seeded into 96-well plates at a density of 1 × 10^4^ cells per well and cultured for various durations (e.g., 24, 48, and 72 hours). MTT stock solution was prepared by dissolving MTT powder in sterile PBS at a final concentration of 5 mg/mL, followed by filtration sterilization and storage in the dark. At each time point, 20 μL MTT solution was added to each well and the plates were incubated for an additional 4 h to allow for the formation of formazan crystals. After incubation, the supernatant was carefully removed without disturbing the attached cells, and 200 μL of DMSO was added to each well to solubilize the formazan crystals. The plates were gently shaken and incubated at room temperature for 10–15 minutes to ensure complete dissolution. Absorbance was measured at 570 nm using a microplate reader. Optical density (OD) values were recorded, and cell proliferation rates were calculated accordingly.


$$\text{Cell Proliferation Rate }(\%)=\frac{OD_{\text{experiment}}}{OD_{\text{control}}}\times 100\%$$

where OD _{experiment}_and OD _{control}_ represent the absorbance of treated cells and untreated control cells respectively.

### 2.7 Cell migration assay

The scratch assay was performed to evaluate the migratory ability of cells. Briefly, cells were cultured in complete medium until they reached 80–90% confluence. Cells were then harvested, counted, and seeded into 6-well plates at a density of 1×10^6^ cells per well. The plates were incubated until a confluent monolayer was formed. A sterile 200 μL pipette tip was used to make a straight scratch in the center of the cell monolayer. Care was taken to maintain consistent scratch width and length across wells. The wells were then gently washed 2–3 times with PBS to remove detached cells and debris. Fresh culture medium was added to each well. Images of the initial wound area were captured under a microscope immediately after scratching (0 h). The plates were then returned to the incubator and cultured for 24 h to allow cell migration. After incubation, images of the wound area were taken again. Wound closure was quantified using ImageJ by measuring the wound area at 0 h and 24h. The percentage of wound closure was calculated to assess cell migration. The percentage of wound closure was calculated as follows:


$$\text{Wound Closure Rate }(\%)=\frac{A_{0}-A_{t}}{A_{0}}\times 100\%$$

where A_0_ represents the initial wound area at 0 h, and A_t_ is the wound area at 24 h. The scratch-wound assay was performed without mitomycin-C or serum starvation. Therefore, wound closure observed after 48 h may partly reflect cell proliferation. Assessment of migration was based on the 24-h data, when proliferative differences between groups were minimal, and should be interpreted cautiously.

### 2.8 Cell invasion assay

Cell invasion ability was assessed using the Transwell assay. Cells were cultured in complete growth medium until reaching 80%–90% confluence. Cells were then harvested, counted, and resuspended in serum-free medium to a final concentration of approximately 3 × 10^4^ cells per well. A total of 300 μL cell suspension was added to the upper chamber of the Transwell insert (8 μM pore size). The lower chamber was filled with medium containing a chemoattractant to promote cell migration. After incubation for 24 hours, non-migrated cells on the upper surface of the membrane were gently removed using a cotton swab. Migrated cells on the lower surface were fixed with methanol for 15–20 minutes, then washed 1–2 times with PBS. The cells were then stained with 0.1% crystal violet solution for 15–30 minutes, followed by thorough rinsing with water. The number of migrated cells was counted under a light microscope.

### 2.9 Colony formation assay

For colony formation assays, cells were cultured in complete growth medium until reaching 80–90% confluence. Cells were then harvested, counted, and seeded into 6-well plates at a density of 200–1000 cells per well. Plates were gently agitated to ensure uniform cell distribution and incubated under standard conditions for 2 weeks. Upon visible colony formation, cells were fixed with pre-chilled methanol for 15–20 minutes, washed with PBS, and stained with 0.1% crystal violet for 15–30 minutes. Excess dye was removed by gentle rinsing. Colonies were subsequently visualized and quantified under a Leica DMi1 microscope, and image analysis software (ImageJ) was employed to assess colony number, size.

### 2.10 Transfection with mRFP-GFP-LC3 adenovirus and fluorescence microscopy

Cells (1×10^4^ per well) were seeded onto coverslips in a 24-well plate. After cells had adhered, they were transfected with mRFP-GFP-LC3 adenovirus and incubated for 12 hours. Following medium replacement, the cells were subjected to a 24-hour treatment of MPA (10 μM). Following fixation with 4% paraformaldehyde and PBS washing, cells were stained with DAPI (BL105A), rinsed with PBS, and mounted using 30% glycerol. Fluorescence microscope (DM IL LED, Leica) was used to examine autophagy, with yellow puncta representing autophagosomes and red puncta representing autolysosome.

### 2.11 Lentiviral infection of cells

IKBKE knockdown lentivirus was obtained from Shanghai Heyuan Biology Co., Ltd. It displayed resistance to purines and had the following sequence: 5′-GCATCATCGAACGGCTAAATA-3′; The NC group sequence of IKBKE shRNA is:5′-CCTAAGGTTAAGTCGCCCTCG-3′. The day prior to transfection, cells were seeded into 24-well plates and allowed to grow overnight, until they had reached 20–30% confluence. After 48 hours, puromycin was introduced into the cells at a final concentration of 8 μg/ml. Cells were maintained under this condition for one week before further experiments.

### 2.12 Statistical analysis

Data analysis was performed using SPSS 16.0. Results are presented as mean ± standard deviation (SD) based on three independent experiments. For the MTT assay, two-way ANOVA was conducted to evaluate the effects of treatment duration (24 h, 48 h, 72 h) and treatment type (drug-resistant, IKBKE knockdown, inhibitor treatment) on cell proliferation. For comparisons between two groups, Student’s t-test was applied. A P value of < 0.05 was considered statistically significant.

## 3 Results

### 3.1 Progestin-resistant cells exhibit increased cell growth

**Figure 1:**
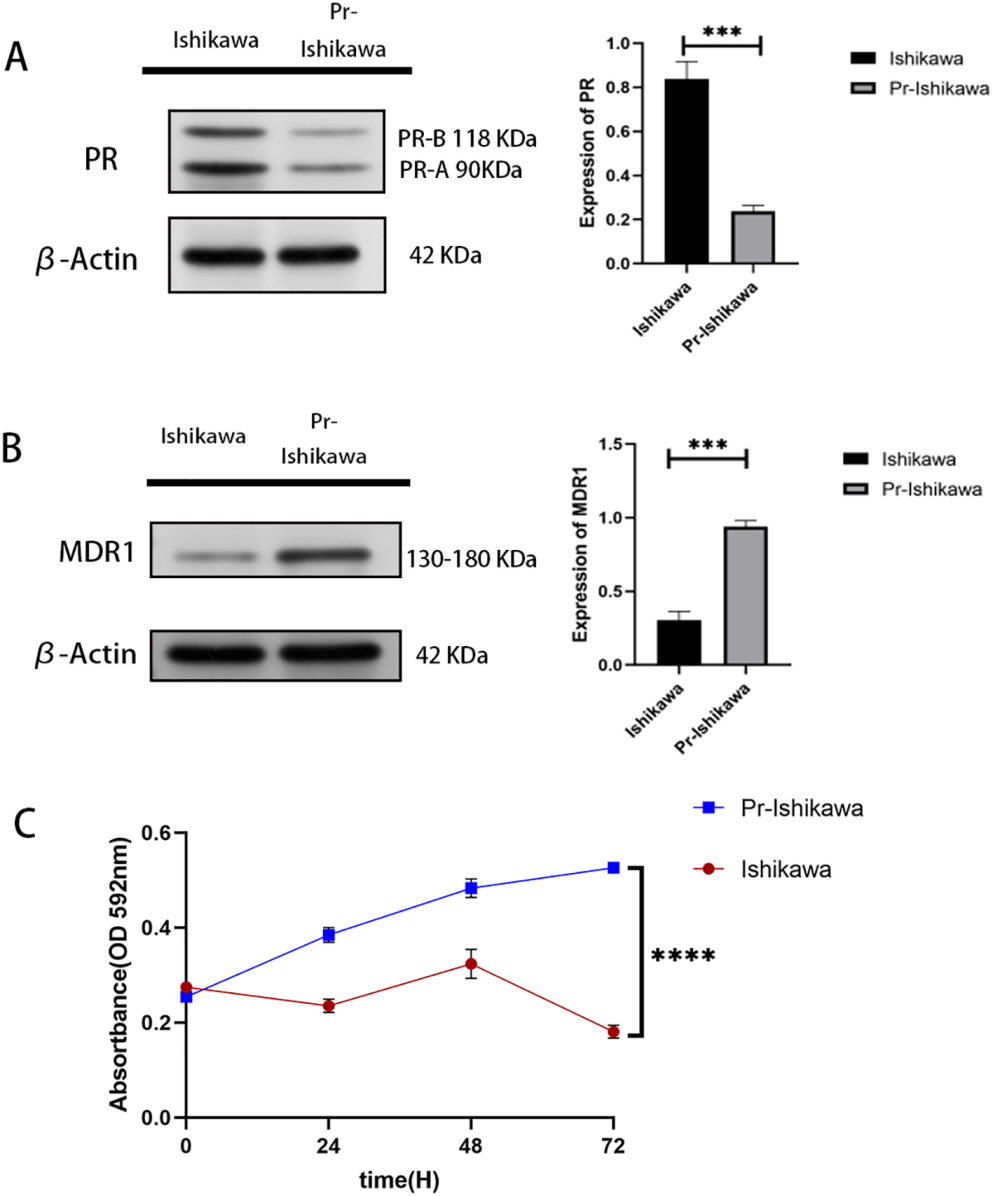
Progestin-resistant cells exhibit increased cell proliferation. **(A)** Western blot analysis of PR expression in progestin-sensitive and progestin-resistant Ishikawa cells. **(B)** Western blot analysis of MDR1 expression in progestin-sensitive and progestin-resistant Ishikawa cells. **(C)** MTT assay showing cell proliferation in response to MPA treatment in progestin-sensitive and progestin-resistant Ishikawa cells. N = 3 independent experiments. The bar graphs are based on the mean ± S.D. ***P<0.001. ****p < 0.0001.

Progestin-resistant EC cell line was established through prolonged exposure to MPA. Western blot analysis confirmed a significant reduction in PR (**Figure 1A**). In addition, multidrug resistance protein 1 (MDR1), a well-established drug resistance marker, was significantly upregulated in progestin-resistant cells (**Figure 1B**).

MTT assay results showed that prolonged progestin exposure induces PR downregulation, sustaining cell proliferation despite continued MPA treatment (**Figure 1C**).

### 3.2 IKBKE inhibits the growth of progestin-resistant EC cells

**Figure 2:**
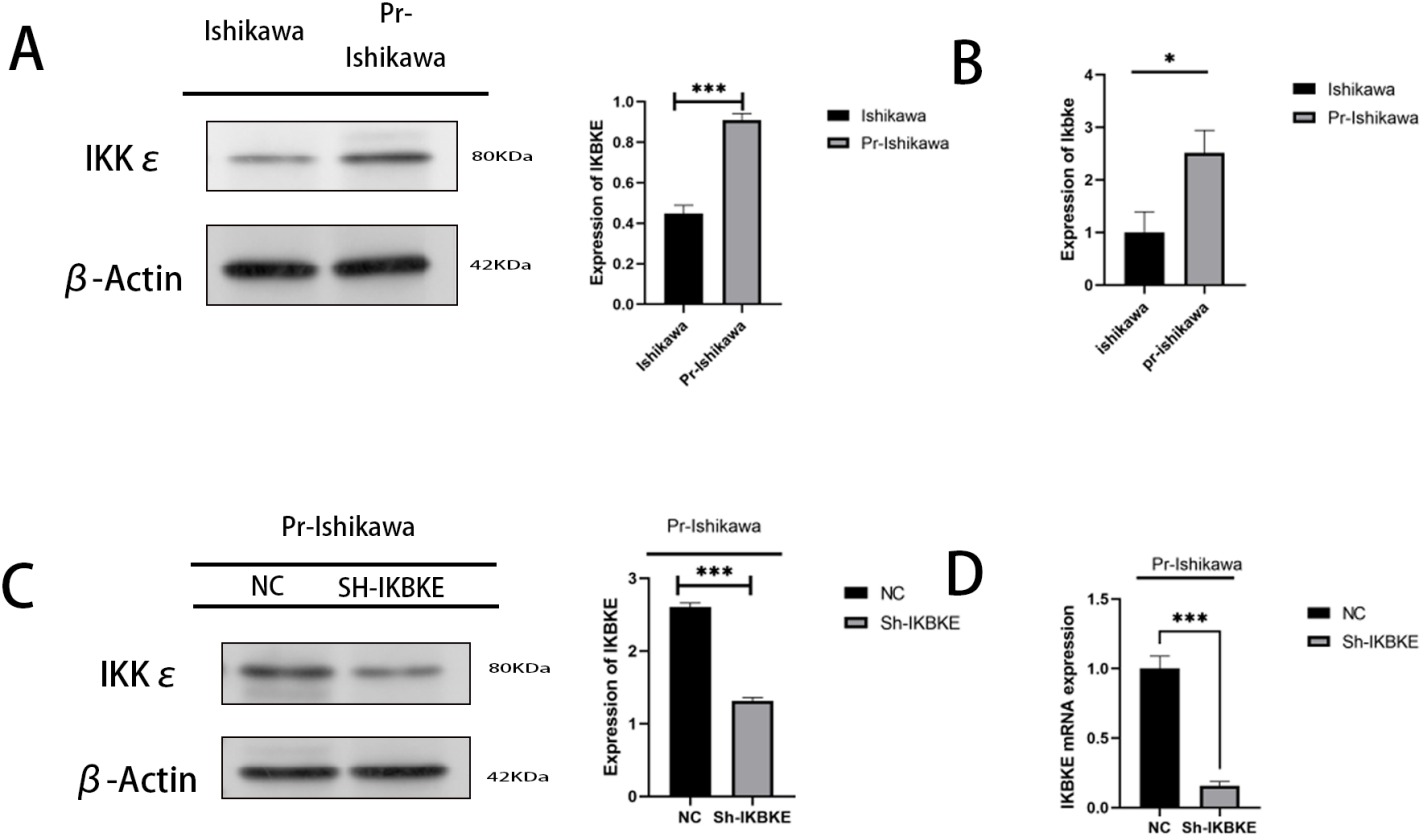
IKBKE expression in progestin-resistant EC cells. **(A, B)** Western blot and RT-qPCR analysis of IKBKE expression in progestin-sensitive and progestin-resistant cells. **(C, D)** Confirmation of IKBKE knockdown by western blot and RT-qPCR in progestin-resistant cells transfected with sh-IKBKE lentivirus. N = 3 independent experiments. The bar graphs are based on the mean ± S.D. *P<0.05. ***P<0.001.

Western blot and RT-PCR assays demonstrated that IKBKE protein and RNA levels were elevated in progestin-resistant EC cells compared to progestin-resistant EC cells (**Figures 2A, B**). Stable IKBKE-knockdown cell lines were generated using shRNA lentivirus transfection, and knockdown efficiency was confirmed via Western blot and RT-qPCR (**Figures 2C, D**).

**Figure 3:**
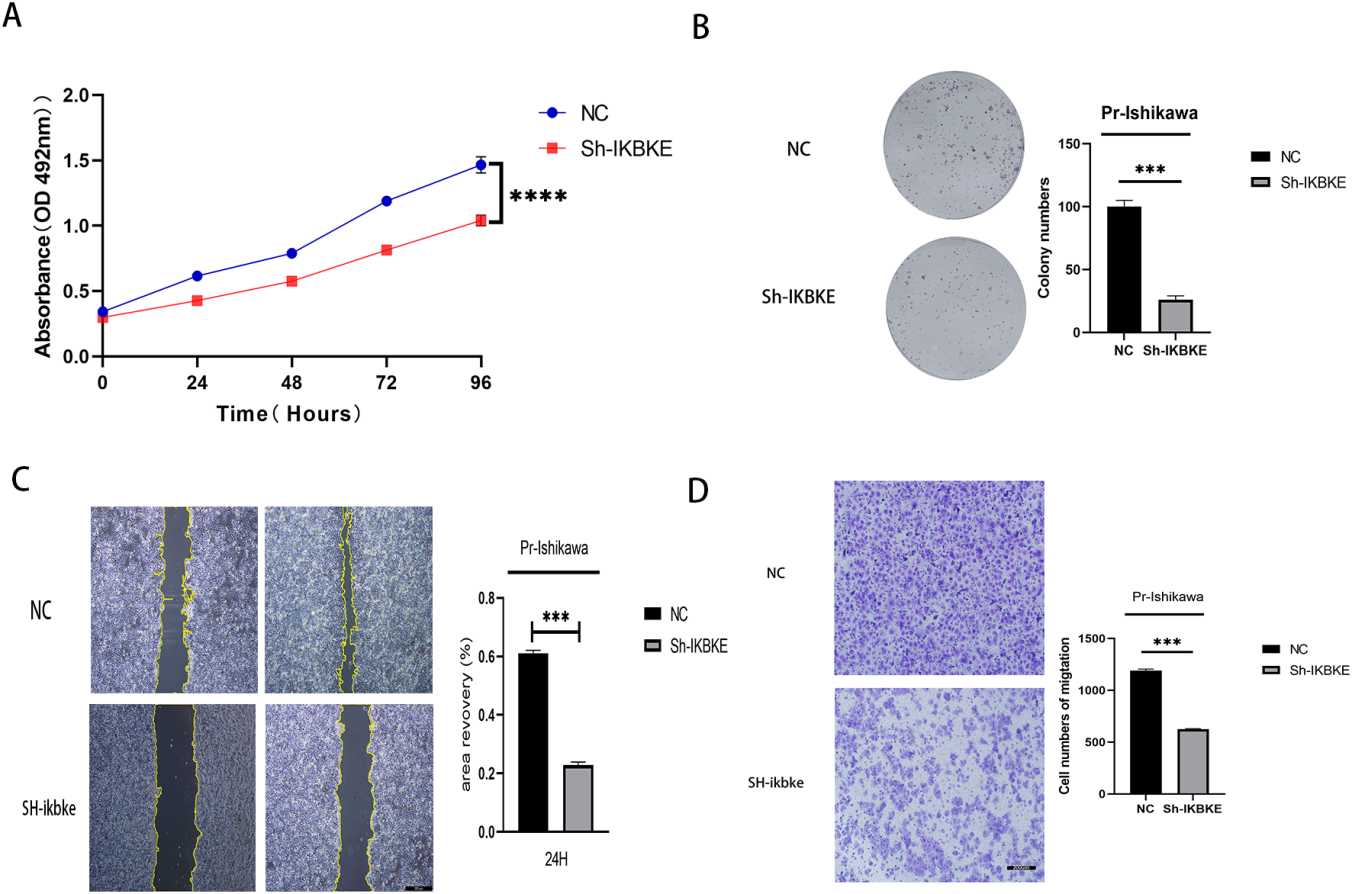
IKBKE knockdown inhibits proliferation and migration in progestin-resistant endometrial cancer cells. N = 3 independent experiments. The bar graphs are based on the mean ± S.D. ***P<0.001. ****p < 0.0001.

The results indicate that knockdown of IKBKE significantly slows down the proliferation of progestin-resistant cells. Specifically, the average OD value of progestin-resistant EC cells with IKBKE knockdown was 0.93, in contrast, untreated progestin-resistant EC cells had an OD value of 1.45(P < 0.0001) (**Figure 3A**). Colony formation assays further demonstrated that at the end of the culture period, progestin-resistant EC cells formed an average of 100 colonies, whereas IKBKE-knockdown progestin-resistant EC cells formed an average of 26 colonies, representing a 47.6% reduction in colony formation (P < 0.0001) (**Figure 3B**).

Wound healing and Transwell assays demonstrated that IKBKE knockdown significantly impaired cell migration. The average wound closure area decreased from 61% to 23% (P<0.001), indicating that IKBKE knockdown significantly impaired cell migration (**Figure 3C**), and the number of migrating cells was reduced from 1,194 to 626 (P< 0.0001), further confirming that IKBKE knockdown significantly reduced both migration and invasion capacities (**Figure 3D**). These findings indicate that IKBKE promotes proliferation and migration in progestin-resistant EC cells.

MTT assay showing reduced proliferation in IKBKE-knockdown cells. (B) Colony formation assay showing significant inhibition of proliferation in IKBKE-knockdown cells. (C) Scratch assay demonstrating a significant demonstrating impaired migration in IKBKE-knockdown cells. (D) Transwell assay revealing that IKBKE knockdown substantially reduces cell migration and invasion. *P<0.05. **P<0.01, ***P<0.001. Scale bar=200 μm.

### 3.3 IKBKE modulates autophagy in progestin-resistant EC cells

**Figure 4:**
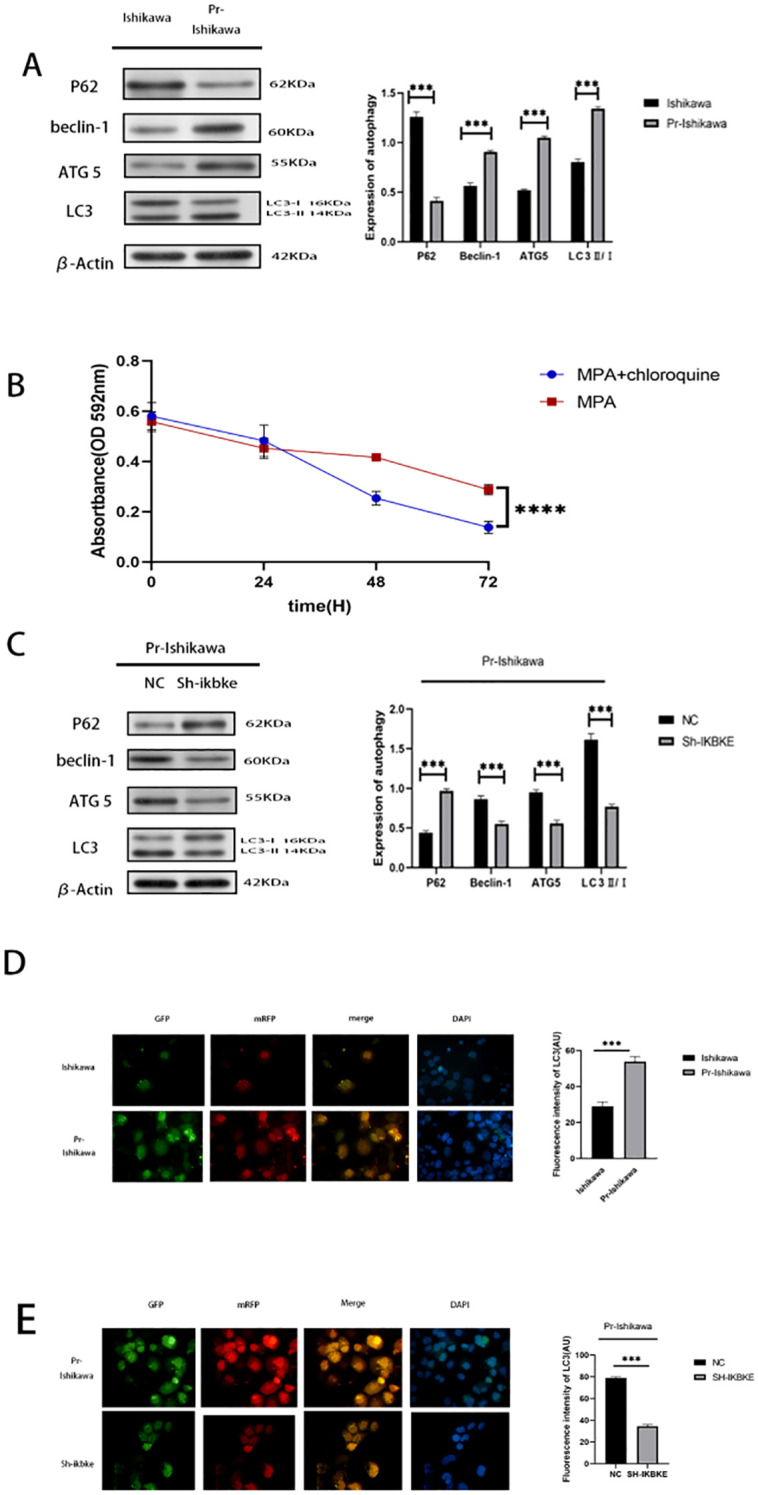
IKBKE modulates autophagy in progestin-resistant endometrial cancer cells. **(A)** Western blot analysis of autophagy markers in progestin-sensitive and progestin-resistant cells. **(B)** MTT assay showing cell proliferative response of progesterone-sensitive and progesterone-resistant Ishikawa cells plus chloroquine to MPA treatment. **(C)** Western blot analysis of autophagy markers following IKBKE knockdown. **(D, E)** Fluorescence microscopy of mRFP-GFP-LC3 adenovirus transfected cells. Green (GFP) indicates autophagosomes, red (mRFP) marks autolysosome, and yellow represents colocalization of GFP and mRFP, indicating intact autophagosomes. Blue (DAPI) stains nuclei. ImageJ was used to select puncta as Regions of Interest (ROIs); average fluorescence intensity was calculated after background subtraction. Fluorescence intensity more precisely captures subtle changes in autophagic activity. N = 3 independent experiments. The bar graphs are based on the mean ± S.D. *P<0.05, ***P<0.001. ****p < 0.0001. Scale bar = 50 μm.

To investigate the role of autophagy in progestin resistance, western blot analysis was performed following MPA treatment to assess the expression of autophagy-related proteins in both Ishikawa cells and progestin-resistant EC cells. Compared with progestin-sensitive EC cells, the resistant cells exhibited elevated expression levels of LC3, Beclin-1, and ATG5, when autophagy is activated, LC3-I undergoes lipidation to convert into LC3-II and binds to autophagosome membranes. Therefore, the LC3-II/β-actin ratio is commonly used as an indicator of autophagy activity. In this study, following IKBKE knockout or CQ treatment, LC3-II band intensity significantly increased while LC3-I band intensity correspondingly decreased. Concurrently, P62 levels markedly decreased, indicating enhanced autophagy activity. (P < 0.01) (**Figure 4A**). To further evaluate whether autophagy contributes to the proliferation of progesterone-resistant EC cells, autophagy inhibitor CQ was administered to progesterone-resistant cells exhibiting increased autophagy. These cells were then co-treated with MPA alongside progesterone-sensitive Ishikawa cells showing reduced autophagy. Subsequently, the proliferation of both cell types was assessed using the MTT assay. In MPA-treated EC cells, the OD value was 0.29, whereas CQ+MPA-treated progestin-resistant EC cells had an OD value of 0.24 (P > 0.5) (**Figure 4B**). These results demonstrate that CQ suppressed proliferation in progestin-resistant EC cells, and that inhibition of autophagy sensitised resistant cells to MPA, resulting in a response comparable to that of progestin-sensitive Ishikawa cells.

To determine whether IKBKE regulates autophagy in resistant cells, Western blot analysis was conducted in progestin-resistant EC cells following IKBKE knockdown. Silencing of IKBKE significantly decreased the expression of LC3, Beclin-1, and ATG5, and increased P62 expression (P < 0.01), indicating inhibition of autophagy (**Figure 4C**).

Further validation was performed using mRFP-GFP-LC3 adenovirus transfection. After MPA treatment, progestin-resistant cells displayed numerous yellow fluorescent puncta, indicating high autophagic flux. In contrast, IKBKE knockdown markedly reduced fluorescence intensity, confirming autophagy inhibition (**Figures 4D, E**). Based on the observed increase in LC3-II and decrease in p62 levels, together with corresponding changes in fluorescence intensity, it can be inferred that IKBKE knockout likely enhances autophagic activity.

### 3.4 CYT387 inhibits proliferation and autophagy in progestin-resistant EC cells

**Figure 5:**
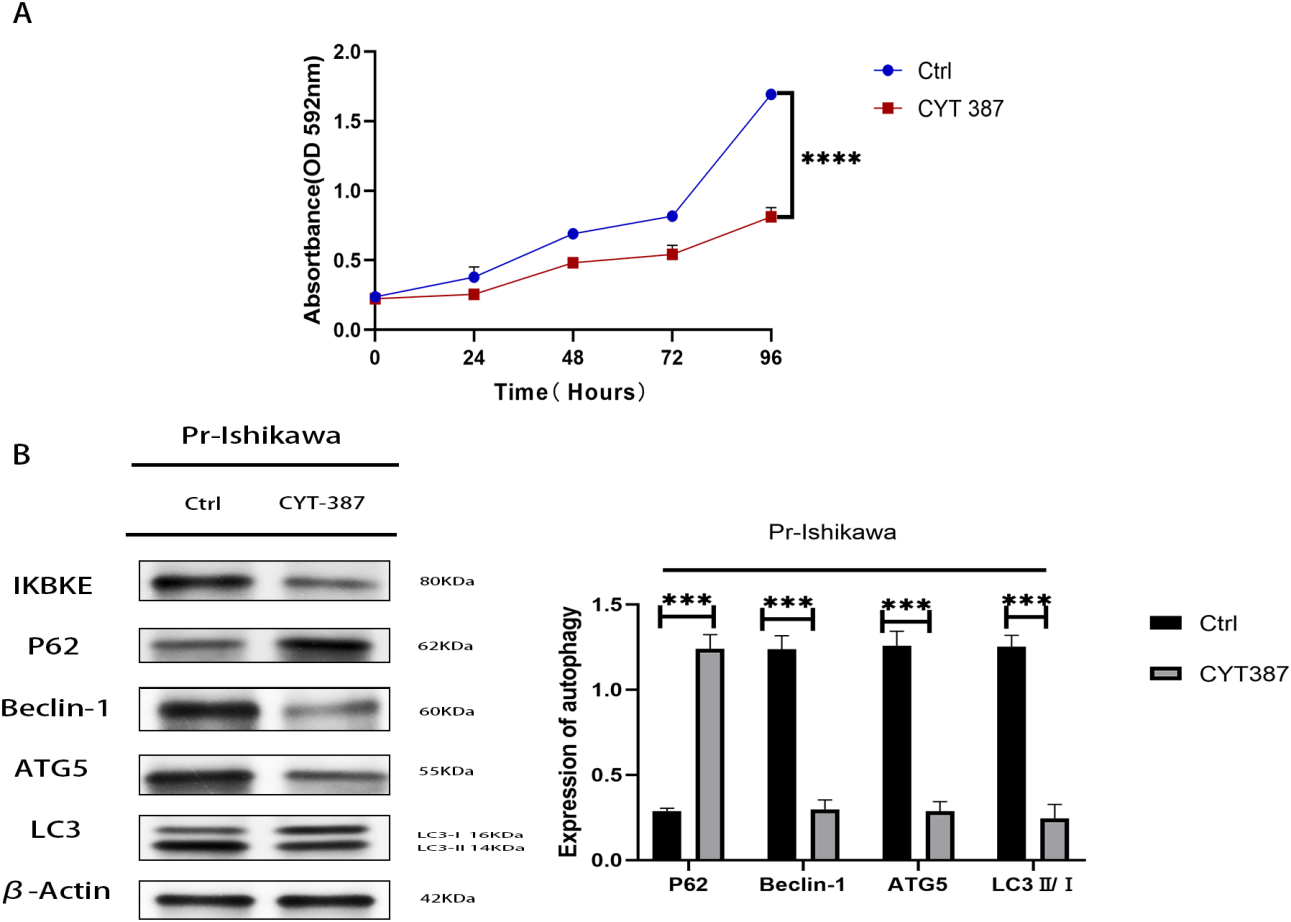
CYT387 suppresses the proliferation and autophagy of progestin-resistant endometrial cancer cells. **(A)** MTT assay showing reduced proliferation following CYT387 treatment. **(B)** Western blot analysis of autophagy markers following CYT387 treatment. N = 3 independent experiments. The bar graphs are based on the mean ± S.D. ***P<0.001. ****p < 0.0001.

To assess the therapeutic potential of CYT387, an IKBKE inhibitor, progestin-resistant EC cells were treated with 534.5 nM CYT387, and cell proliferation was evaluated using MTT assays. CYT387 treatment significantly reduced cell proliferation (p<0.0001), with the average OD value of CYT387-treated progestin-resistant EC cells at 0.66, corresponding to a proliferation rate of 37.5% ± 7.4%. In contrast, untreated progestin-resistant EC cells had an OD value of 1.64, corresponding to a proliferation rate of 223.7% ± 16.9% (p<0.001) (**Figure 5A**). Western blot analysis revealed that CYT387 treatment also suppressed autophagy, as indicated by a significant decrease in LC3B-II(LC3B/β-actin), Beclin-1, and ATG5 levels and an increase in P62 expression (**Figure 5B**). These results suggest that CYT387 effectively inhibits both proliferation and autophagy in progestin-resistant EC cells, highlighting its therapeutic potential in EC.

## 4 Discussion

EC is a common malignant tumor in women. Despite the availability of progestin-based therapies, resistance remains a significant challenge, compromising therapeutic efficacy and fertility preservation (31). Understanding the mechanisms underlying progestin resistance in EC is essential developing more effective treatment strategies. In the present study, we demonstrated thatIKBKE plays a crucial role in mediating progestin resistance in EC. Specifically, IKBKE knockdown significantly inhibited the proliferation and migration of progestin-resistant EC cells, suggesting its potential as a therapeutic target.

Previous studies have shown that IKBKE is associated with inflammatory responses and metabolic diseases and is highly expressed in various malignant tumors, including non-small cell lung cancer, renal clear cell carcinoma, breast cancer, ovarian cancer, and glioma (15). Its expression is positively correlated with tumor grade, drug resistance, and malignant progression. For instance, Lee found elevated IKBKE levels in 63 of 96 ovarian cancer specimens, which correlated with advanced and high-grade tumors. Furthermore, IKBKE overexpression was linked to cisplatin resistance, while knockdown of IKBKE reversed this resistance (32). Notably, IKBKE has emerged as a therapeutic target for advanced prostate cancer due to its role in inhibiting resistance to androgen receptor-targeted therapies, as well as its effects on proliferation, migration, and colony formation (33–35). Consistently, our findings indicate that IKBKE knockdown reduces proliferation and migration in progestin-resistant EC cells, highlighting its role in sustaining drug resistance and tumor progression.

Our study revealed that in progesterone-resistant cells, elevated IKBKE expression was accompanied by increased levels of autophagy-related proteins (such as LC3, Beclin-1, and ATG5) and decreased P62 expression, indicating enhanced autophagy activity. Conversely, IKBKE knockdown suppressed autophagy, manifested by reduced LC3, Beclin-1, and ATG5 levels and increased P62 expression. Given autophagy’s role as a survival mechanism enabling cancer cells to adapt to metabolic stress and evade treatment-induced apoptosis (25), these findings align with previous studies demonstrating that IKBKE functions as a positive regulator of autophagy, facilitating tumor progression and therapy resistance (36, 37). However, autophagy plays a dual role in cancer, functioning as both a pro-survival and pro-death mechanism depending on the context. While our results indicate that autophagy supports drug resistance in progestin-resistant EC cells, some studies suggest that sustained autophagic activity can promote tumor cell death (38). This discrepancy may stem from differences in progestin receptor expression, tumor microenvironmental factors, or alternative regulatory mechanisms. Further investigation is warranted to elucidate the precise role of autophagy in progestin-resistant EC and determine whether autophagy inhibition or induction is the optimal therapeutic approach. Precise quantification of autophagy flux requires further validation through co-treatment experiments with BafA1.

We also explored the therapeutic potential of CYT387, a selective IKBKE inhibitor. CYT387 has been tested in renal cell carcinoma, myeloproliferative necrosis, and myeloproliferative tumors (39, 40). In renal cell carcinoma, the combination of CYT387 and dasatinib decreased cell proliferation and increased apoptosis. In non-small cell lung cancer resistant to epidermal growth factor receptor inhibitors, the combination of cetuximab and CYT387 significantly inhibited proliferation (41). Our study shows that CYT387 effectively reduces IKBKE expression, suppresses autophagy, and inhibits the proliferation of progestin-resistant EC cells. These findings suggest that targeting IKBKE with CYT387 may serve as a viable therapeutic strategy for progestin resistance in EC.

At present, the precise mechanisms underlying progestin resistance in EC remain incompletely understood. One key pathway implicated in drug resistance across various gynecological malignancies, including EC, cervical cancer, and ovarian cancer, is the PI3K/Akt/mTOR signaling pathway (42). Dysregulated activation of this pathway prevents drug-induced growth inhibition, leading to sustained proliferation and survival. Amplification and overexpression of PI3K and Akt have been observed in EC, reinforcing this pathway as a potential therapeutic target. Notably, sustained activation of PI3K/Akt signaling in drug-resistant EC strains has been linked to a failure of drug-mediated cell cycle arrest and apoptosis, further contributing to resistance. Increasing PTEN expression through protease inhibitors has been shown to restore drug sensitivity in these resistant strains (43). In addition to the PI3K/Akt/mTOR pathway, progesterone may also promote cell proliferation by activating membrane receptors, which not only enhance the expression of proliferation-related genes but also inhibit apoptotic pathways, including TANK, NF-κB, Bcl-1, and Bcl-2 (44, 45). Given that IKBKE can activate NF-κB and regulate autophagy, it may serve as a critical mediator of progesterone-driven proliferation and survival signaling. Future studies should investigate the interplay between IKBKE, NF-κB activation, and autophagy in progestin-resistant EC to refine therapeutic strategies.

This study has several limitations. First, our findings are based on a single progestin-resistant EC cell line, and validation using additional cell lines would strengthen the generalizability of our conclusions. Second, further exploration of downstream signaling pathways influenced by IKBKE would provide deeper mechanistic insights into its role in progestin resistance. This study evaluated cell migration only and did not assess invasion (which requires Matrigel). The regulatory effect of IKBKE on endometrial cancer cell invasion requires subsequent experimental validation. Lastly, this study focused on the *in vitro* function of IKBKE using the Ishikawa cell line model. No *in vivo* experiments, such as subcutaneous xenografts, were performed to evaluate the effects of IKBKE knockdown on tumor growth, pharmacological response, or Ki-67/LC3-II expression in tumor tissues. Therefore, the conclusion that IKBKE represents a therapeutic target should be considered a preliminary observation derived from *in vitro* findings, *in vivo* studies are needed to assess the therapeutic potential of IKBKE inhibition.

## 5 Conclusions

Our study demonstrates that progestin-resistant EC cells exhibit increased proliferation and migration, driven by IKBKE overexpression. Inhibition of IKBKE significantly impairs these tumorigenic properties while reducing autophagic activity. Furthermore, the selective IKBKE inhibitor CYT387 effectively suppresses both cell proliferation and autophagy in progestin-resistant EC cells. These findings provide novel insights into the role of IKBKE in mediating progestin resistance and lay the groundwork for the development of targeted therapeutic strategies to enhance treatment efficacy in EC.

## Data Availability

The raw data supporting the conclusions of this article will be made available by the authors, without undue reservation.
